# Infectious Disease and Its Notification

**Published:** 1916-02-19

**Authors:** 


### The Hospital

The Workers' Newspaper of Administrative Medicine and Institutional Life, Administration, National Insurance and Health.

No. 1549, Vol. LIX. SATURDAY, FEBRUARY 19, 1916. PRICE ONE PENNY.

### INFECTIOUS DISEASE AND ITS NOTIFICATION.

The domestic affairs of the medical profession have of recent months been so much entangled with the relations of the profession to the Army that questions which might otherwise have engaged attention have been allowed to pass with little or no notice. One of these is the responsibility of practitioners under the Notification of Infectious Diseases Act. Partly by the action of the Local Government Board and partly by the decisions of various local health authorities, the operations of this Act have been much extended so that the list of diseases to which its provisions apply is now much more comprehensive than the original catalogue sanctioned by Parliament. The law of the land, of course, stands beyond challenge, and no one disputes the legal obligation which falls upon the practitioner when he finds himself in contact with a patient suffering from one or other of the diseases authoritatively declared to be "infectious." It is plainly his duty to forward as the law demands the required notification to the local Medical Officer of Health. Nor is there any question that, speaking generally, it is in the interest of the community that the occurrence of such diseases should be made known to the local health authority. Yet while these two propositions are admitted it is open to question whether the method enacted by Parliament is the one best fitted to attain the desired end, and most certainly there are attached to it conditions which not very infrequently place the medical practitioner in a position of considerable difficulty. The subject is of present importance seeing that the area of "notification" is always tending to become enlarged, and in view also of the opinions which were expressed by some of the speakers in a recent discussion at the General Medical Council.

The first comment which may be made upon the present rule is that it places the practitioner under the stress of divided duties and possibly of conflicting duties. Clearly his first care is the welfare of the patient; it is to this end he has been summoned, and for its discharge he accepts his fee. Along with this obligation is the honourable claim that he shall keep silence in reference to his patients' illness and affairs. On the other hand is the demand of the law that certain information which but for this demand it would be shameful to disclose shall be formally announced to the public authorities. The law must be obeyed; of this there is no question. Yet for our own part we have always regretted that it was deemed necessary to make the medical practitioner the direct agent by which notification is to be effected. It is a matter of high importance that patients should have unlimited confidence in the honourable secrecy of their medical advisers, and whatever tends to weaken this confidence is harmful to the public interest. Even when the breach is committed under legal pressure it carries some measure of lessened trust and so far impairs what it is a general interest to promote. Had the duty of notification been thrown upon the relatives or guardians of the patient, when the nature of the illness had been duly certified to them by the medical practitioner, the invasion of the confidential relations between doctor and patient would have been avoided, and this without prejudice to the general purpose of the Legislature. In saying this, however, we fully recognise that we are reviving a lost cause. For good or ill the decision was taken years ago and the principle of it has received further application, as, for example, in the Notification of Births Act. The law compels the practitioner, however distasteful to him may be the task, to reveal to the public authorities information which he has received while acting in the capacity of the patient's private medical adviser.

A second point where some conflict of interest arises is the date at which notification should be effected, and it is here that some exception may be taken to certain expressions uttered by certain members of the General Medical Council. Clearly it is in the interest of the patient that his case shall not be certified to the health authorities so long as there remains any shadow of doubt in reference to the nature of the illness from which he is suffering. Notification will mean the invasion of his house by public officials, interference with his business or other interests, and possibly his removal to hospital. On the other hand, and with equal clearness, it is in the interest of the health authorities to obtain notification of every case at the earliest possible date. Thus the medical practitioner stands under the pressure of two opposing claims, and a wrong decision in either direction is likely to bring him trouble. What the law requires is that when he is " of opinion " that such-and-such disease is present, he must forward the required certificate, and not unnaturally the tendency of the health authorities is to expect this " opinion " when there is what may be called a mere balance of evidence; while, as already pointed out, the interest of the patient calls for the complete exclusion of all reasonable doubt. Hence it is not enough to say, as was said in the discussion above mentioned, that the practitioner has " a duty towards the State," and that " early notification is in the interest of the public welfare." Let these propositions be allowed, but it yet remains true that the practitioner has a duty towards the patient from whom he receives both confidence and payment. Only in a very narrow sense is the practitioner a State officer, while in the fullest possible degree is he the private and confidential adviser of the individual of whose interests he has accepted the charge. Further, if in his anxiety to meet the public claim he hurries notification short of certainty, and time and the event show him to be in error, he is very soon made to realise that though engaged on the service of the State he enjoys no measure of State protection. On the contrary, he is allowed to bear the full brunt of the mistake, and this certainly includes loss of reputation and possibly also may include a direct financial penalty. In these circumstances it is not surprising that practitioners find evidence which is sufficient to justify suspicion and the taking of precautions quite insufficient for the confident and specific " opinion " which notification demands. The situation could often be eased by some administrative flexibility on the part of the public health authorities, but its inherent vice could only be removed by legislation, and this in the present circumstances is out of the question. The fault lies not with the family practitioner, but with the conditions which have been imposed upon him by Act of Parliament.

